# Benchmarking transformer-based models for medical record de-identification in a single center multi-specialty evaluation

**DOI:** 10.1016/j.isci.2025.113732

**Published:** 2025-10-08

**Authors:** Rachel Kuo, Andrew A.S. Soltan, Ciaran O’Hanlon, Alan Hasanic, David A. Clifton, Gary Collins, Dominic Furniss, David W. Eyre

**Affiliations:** 1 Nuffield Department of Orthopaedics, Rheumatology, and Musculoskeletal Sciences, University of Oxford, Oxford OX3 7LD, UK; 2 Oxford University Hospitals NHS Foundation Trust, Oxford OX3 9DU, UK; 3 Department of Oncology, University of Oxford, Oxford OX3 9DY, UK; 4 Nuffield Department of Primary Care Health Sciences, University of Oxford, Oxford OX2 6GG, UK; 5 Department of Engineering Science, University of Oxford, Oxford OX1 2JD, UK; 6 Oxford Suzhou Centre for Advanced Research, University of Oxford, Suzhou, Jiangsu 215123, China; 7 Centre for Statistics in Medicine, University of Oxford, Oxford OX3 7LD, UK; 8 United Kingdom EQUATOR Centre, Oxford OX3 7LD, UK; 9 Big Data Institute, Nuffield Department of Population Health, University of Oxford, Oxford OX3 7LF, UK; 10 NIHR Oxford Biomedical Research Centre, Oxford OX3 9DU, UK; 11 Health Protection Research Unit in Healthcare Associated Infections and Antimicrobial Resistance, Oxford OX3 7LF, UK

**Keywords:** Health informatics, Artificial intelligence

## Abstract

Protecting patient confidentiality is central to enabling research using electronic health records. Automated text de-identification offers a scalable alternative to manual redaction. However, different approaches vary in accuracy and adaptability. We evaluated four transformer-based, task-specific models and five large language models on 3,650 clinical records spanning general and specialty datasets from a UK hospital group. Records were dual-annotated by clinicians, allowing precise comparison of performance. The Microsoft Azure de-identification service achieved the highest F1 score, approaching clinician performance, while fine-tuned AnonCAT and GPT-4-0125 with few-shot prompting also performed strongly. Smaller LLMs frequently over-redacted or produced hallucinatory content, limiting interpretability. Task-specific models demonstrated greater stability across datasets, while low-level adaptation improved performance in both model classes. These findings highlight that automated de-identification systems can provide effective support for large-scale sharing of clinical records, but success depends on careful model choice, adaptation strategies, and safeguards to ensure robust data utility and privacy.

## Highlights

• Azure de-identification achieved near-human performance (F1 0.939)
• Task-specific models were more stable; GPT-4 excelled with few-shot input
• Smaller LLMs often over-redacted or hallucinated, harming record utility
• Adaptation boosted accuracy but deployment needs safeguards and governance

## Graphical abstract

**Figure:**
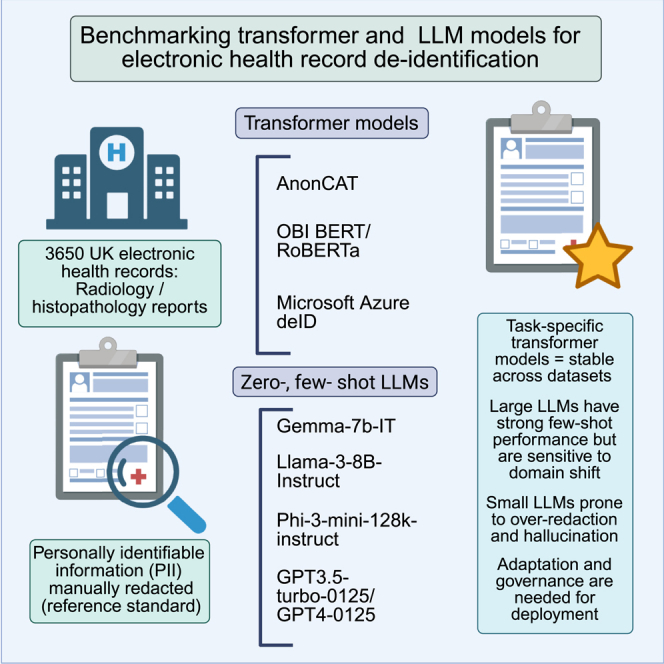

## Introduction

Routinely collected healthcare data for millions of patients are stored within electronic health records (EHRs) to facilitate care delivery and efficient communication between professionals.^1^^,^^2^ These data are diverse and include structured data, such as laboratory results, and semi-structured or unstructured information, such as diagnostic reports. The resulting data-store is a valuable resource for research, audit, education, and quality improvement.^3^ Recently, there has been an increasing interest in utilizing EHR data to develop and validate deep learning models to improve clinical outcomes and care efficiency.^4^

Sharing data for secondary use necessitates robust de-identification to preserve confidentiality. Identifiers, such as patient names, must be removed to minimize the risk of re-identification, comply with data protection legislation, and establish public trust and acceptability.^5^^,^^6^ In Europe, General Data Protection Regulation defines personal data as any information that relates to an identified or identifiable individual. In the US, identified healthcare records are personally identifiable information (PII) when they contain any of 18 identifiers defined in the Health Insurance Portability and Accountability Act.

Effective redaction of PII from semi- or un-structured medical records can be challenging. Manual de-identification, in which humans review and redact personal information, is time-consuming and costly, limiting the scale of data that can safely be made available.^7^ Automated de-identification offers considerable benefits to hospitals, researchers, and patients. However, some categories of PII, such as names and locations are poorly recognized using regular expressions or rule-based algorithms, requiring curation of extensive local dictionaries to ensure adequate redaction.^8^^,^^9^^,^^10^ Rule-based models may be prone to over-redaction or fail to account for nuanced differences in regional formats, compromising the utility of de-identified data.^8^^,^^9^ Strategies relying on local dictionaries are vulnerable to spelling errors and require re-definition if applied to new datasets.^10^ Additionally, there is heterogeneity in clinical language across medical specialties and sites, necessitating domain adaptation to maintain performance.

Domain-agnostic automated de-identification that requires no or minimal adaptation could facilitate safe and cost-effective data sharing at scale. Recently, transformer-based natural language processing (NLP) models have shown promise in de-identification without the requirement for domain-specific adaptation.^9^^,^^11^^,^^12^ Existing research shows that large language models (LLMs) may be able to perform clinical/biomedical NLP tasks with no domain-specific training (zero-shot inference), or with a few examples of input and desired output (few-shot learning).^9^^,^^13^^,^^14^ However, it is unclear how large generalist models perform in comparison to task-specific models, which are often designed to run on premise and may include vendor support for EHR integration. Moreover, some task-specific models permit adaptation and fine-tuning, but the quantitative benefit of this for de-identification is not yet clear. LLMs may provide a lower barrier to use, particularly when zero- or few-shot strategies can be applied without requiring large, labeled datasets, or extensive computational resources. A critical challenge of LLMs is the phenomenon of “hallucination,” in which LLMs generate erroneous output, such as clinical opinions or meta-commentary (e.g., “you have asked me to redact this record”).^15^^,^^16^^,^^17^ These outputs may compromise the interpretability of de-identified records or introduce misleading information. Understanding the comparative strengths and limitations of each may guide real-world deployment.

In this study, we evaluated four existing proprietary de-identification software tools, and five LLMs in the task of text de-identification, using no and minimal adaptation strategies and a new dataset of 3,650 mixed clinical records from a group of National Health Service (NHS) hospitals in the United Kingdom. Existing public benchmark datasets and associated de-identification challenges, including i2b2, MIMIC-II, and n2c2, are derived from the US healthcare systems, and largely consist of discharge summaries, progress notes, and nursing documentation.^18^^,^^19^^,^^20^ Our dataset introduces several key differentiators: first, it reflects UK-specific identifiers and naming conventions, enabling international comparison with US-based benchmarks. Second, it consists of radiology and histopathology records: document types that are less represented in existing benchmarks, yet central in multi-modal research, such as those focusing on diagnosis using computer vision. These documents vary in structural and linguistic complexity and include domain-specific terminology such as rare, eponymous fracture patterns. These features offer a distinct evaluation setting for assessing the generalizability and robustness of de-identification models, complementary to established US-based benchmarks.

## Results

### Dataset description

We labelled data using a modified ontology based on HIPAA PII categories (STAR Methods, Table 1).^21^ We analyzed 3,650 records consisting of 479,760 words, of which 17,496 (3.65%) were PII. Record length and PII prevalence differed across datasets (Table S1). The most frequent forms of PII were names (6,901/17,496, 39.4%), of which the majority were healthcare professional names (6,870/6,901, 99.6%) (Table S2). In common with previous research, only a minority of names were patient names (31, 0.4%).^8^

The next most frequent PII categories were “other unique identifiers” (4,758, 27.2%; comprising professional details, names of external healthcare organizations, and names of hospitals or healthcare units), dates (3,641, 20.8%), medical record numbers (1,408, 8.0%), and telephone numbers (334, 1.9%). All other PII categories had a prevalence of <1%. There were no occurrences of fax numbers, health plan beneficiary numbers, vehicle/device identifiers, or IP addresses.

### Inter-annotator results

There was excellent agreement between clinician annotators. Pairwise-F1 for classification of PII/non-PII was 0.977 (0.957–0.991), precision 0.967 (0.932–0.993), and recall 0.986 (0.971–0.997) (Table S3). Character-level Cohen’s Kappa was 0.895 (95% CI, 0.886–0.904), signifying high consistency in how PII was delineated by clinician annotators. All discrepancies between annotators were due to cases in which one annotator did not notice a PII-word. Once identified, there were no disagreements about whether a word should be classified as PII. The bilingual evaluation understudy (BLEU) score between the original, unredacted records, and manually redacted records was 0.931 (0.923–0.934), reflecting the prevalence of PII in the dataset (Table 2). The Levenshtein distance was 67.0 (63.3–70.8).

**Table 2:** String similarity between original and redacted text

Model type	Model name	Number of shots	BLEU score (95% CI)	Levenshtein distance (95% CI)
Inter-annotator		N/A	0.931 (0.923–0.934)	67.0 (63.3–70.8)
Proprietary de-identification software	Microsoft Azure de-identification service	N/A	0.929 (0.927–0.932)	35.7 (34.1–37.6)
	AnonCAT	Base model	0.948 (0.946–0.950)	30.2 (28.8–31.7)
		Adapted and fine-tuned	0.929 (0.926–0.931)	44.0 (41.8–46.0)
	obi/deid_roberta_i2b2	N/A	0.932 (0.930–0.933)	31.6 (30.6–32.6)
	obi/deid_bert_i2b2	N/A	0.932 (0.931–0.933)	34.1 (33.0–35.2)
Large language models	Gemma-7b-IT	0	0.071 (0.068–0.075)	749.3 (726.1–773.9)
		1	0.032 (0.030–0.035)	860.4 (838.1–883.8)
		5	0.025 (0.023–0.026)	758.2 (735.6–781.6)
		10	0.031 (0.029–0.033)	741.8 (717.1–765.33)
	Llama-3-8B-Instruct	0	0.259 (0.250–0.269)	465.0 (448.7–482.2)
		1	0.500 (0.491–0.510)	145.2 (139.4–151.0)
		5	0.769 (0.760–0.778)	71.3 (67.2–75.1)
		10	0.693 (0.682–0.703)	106.0 (99.3–113.5)
	Phi-3-mini-128k-instruct	0	0.396 (0.383–0.407)	836.3 (790.6–882.7)
		1	0.515 (0.498–0.533)	603.8 (566.2–640.0)
		5	0.482 (0.468–0.497)	384.8 (369.0–400.3)
		10	0.663 (0.649–0.676)	281.4 (262.4–302.1)
	GPT-3.5-turbo-0125	0	0.838 (0.832–0.843)	87.6 (83.9–92.1)
		1	0.878 (0.873–0.883)	73.0 (69.4–77.2)
		5	0.926 (0.921–0.930)	51.6 (47.9–56.2)
		10	0.932 (0.928–0.936)	47.2 (41.1–52.1)
	GPT-4-0125	0	0.920 (0.915–0.924)	50.5 (46.5–55.3)
		1	0.922 (0.917–0.927)	51.4 (47.3–56.1)
		5	0.925 (0.921–0.929)	54.3 (49.7–59.2)
		10	0.920 (0.915–0.924)	52.6 (48.4–57.3)

Per model BLEU scores and Levenshtein distances are shown.

### Model results

#### PII vs. non-PII

**Figure 1:**
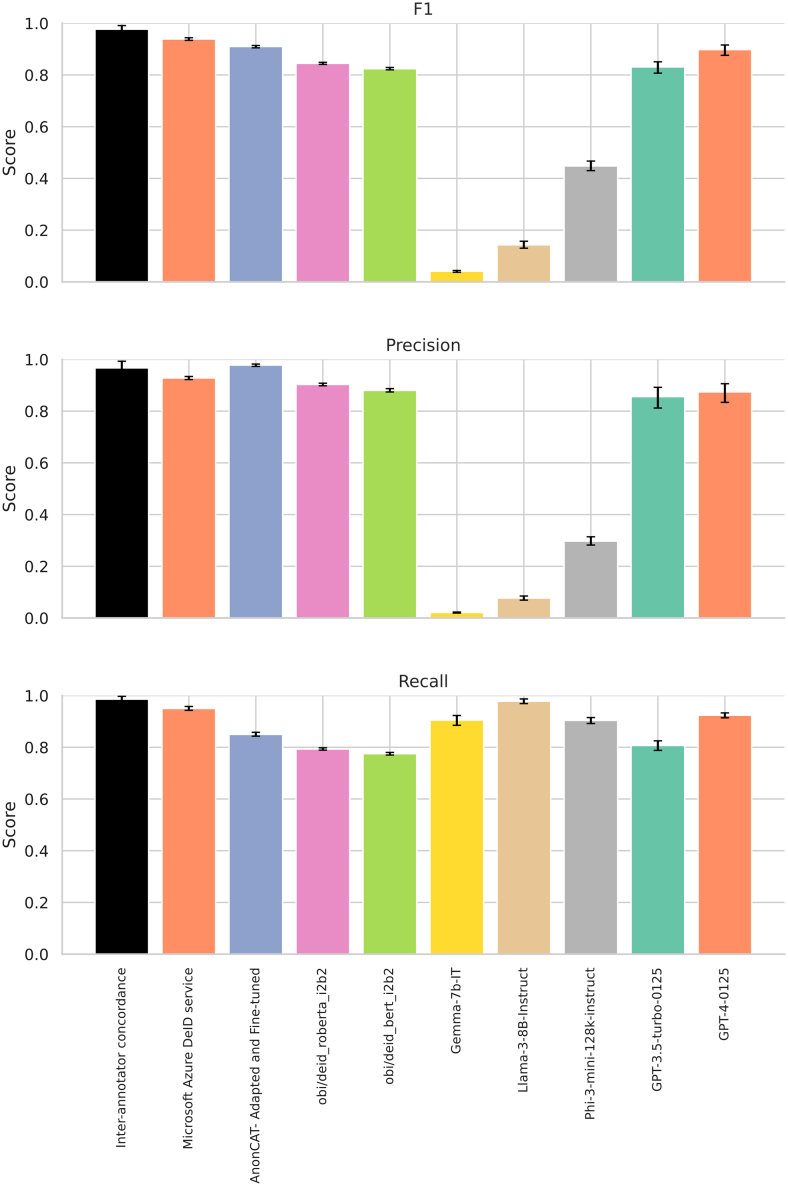
Model performance, for PII vs. non-PII classification, with 95% confidence intervals All LLM results are plotted using performance with ten shots. FT-AnonCAT performance is plotted with the requirement to redact healthcare professional titles. Panel A shows F1 score, panel B recall, and panel C precision. Data are represented as mean ± 95% confidence intervals.

There was substantial variation in performance between the evaluated models (Figure 1; Table S3). The Microsoft Azure de-identification service had the highest F1 score 0.939 (95% CI, 0.934–0.944), precision of 0.928 (0.922–0.934), and recall of 0.950 (0.943–0.958), approaching clinician performance. The fine-tuned, concept-expanded AnonCAT (FT-AnonCAT) model had an F1 score of 0.910 (0.905–0.914), precision of 0.978 (0.973–0.982), and recall of 0.850 (0.843–0.858), including the redaction of healthcare professional titles as this was added during the adaptation process.

The OBI RoBERTa i2b2 model outperformed the OBI BERT i2b2 model, possibly reflecting strengths of the underlying RoBERTa model’s training procedure, including a larger training corpus and batch size, dynamic masking procedure, and improved hyperparameter optimization. OBI RoBERTa i2b2 outperformed GPT-3.5-turbo-0125 with 10 shot prompting as measured by F1 score, but was surpassed by GPT-4-0125.

The best performing LLM was GPT-4-0125 with ten-shot learning, with F1 score of 0.898 (0.876–0.915), precision of 0.874 (0.834–0.906), and recall of 0.924 (0.914–0.933; Figure S1). This was followed by GPT-3.5-turbo-0125 with ten-shot learning, with F1 score of 0.831 (0.807–0.851), precision of 0.856 (0.812–0.892), and recall of 0.807 (0.788–0.825).

There was improvement in GPT-3.5-turbo-0125 with few-shot learning: the F1 score rose from 0.530 (0.514–0.547) at zero shots to 0.831 (0.807–0.851) with 10 shots, driven by improved precision; recall remained similar through all iterations of zero- and few-shot learning. On qualitative examination with none, or fewer in-context examples, the LLM over-redacted records, including clinically relevant information such as diagnosis, or details of pathology.

The performance of other LLMs was more modest. The next best was Phi-3-mini-128k-instruct, also improving with few-shot learning, with F1 score 0.146 (0.140–0.153) at zero-shots, improving to 0.448 (0.430–0.467) with ten-shots. However, there was significant imbalance between precision and recall. At ten-shots, precision was 0.297 (0.282–0.314) and recall 0.904 (0.892–0.915). This was consistent with our findings on qualitative examination, showing over-redacted records.

Llama-3-8B-Instruct showed the same pattern of over-redaction, showing best performance at five shots, with an F1 score of 0.198 (0.181–0.216), precision of 0.077 (0.069–0.085), and recall of 0.990 (0.983–0.995). We did not observe any change in performance from zero-to few-shot learning with Gemma-7b-IT. The F1 at zero shots was 0.089 (0.086–0.092) and at ten shots 0.041 (0.037–0.044). At ten shots, precision was 0.021 (0.019–0.023) and recall was 0.905 (0.885–0.923). Qualitative examination of Gemma-7b-IT output showed hallucinatory content was universally present.

#### Evaluation of text similarity and LLM hallucinations

BLEU scores were high for all four task-specific models, reflecting close text similarity post redaction to the original text (Table 2). This was similar to the BLEU score recorded between clinician redaction and reference text (Table 2). The Levenshtein distances for all task-specific models were low, and slightly lower than the distance reported for clinician redaction, in keeping with high similarity between output and reference texts at the character level. Qualitative examination of the outputs showed no evidence of hallucination, and therefore that the lower distance likely reflects performance characteristics of the models.

The best performing LLMs, GPT-4-0125 and GPT-3.5-turbo-0125, had consistently similar BLEU scores and Levenshtein distances to values recorded for clinician redaction, and showed no evidence of hallucination on qualitative examination.

Both Phi-3-mini-128k-instruct and Llama-3-8B-Instruct showed improved BLEU scores and Levenshtein distances across zero- and few-shot learning. On qualitative examination, we confirmed that both Phi-3-mini-128k-instruct and Llama-3-8B-Instruct showed evidence of hallucinatory behavior at zero-, one- and five-shot learning. This included explanations of the task or output (e.g., “this text does not contain explicit identifiers, therefore the text remains unchanged”) alongside nonsensical string (e.g., long spans of punctuation). We did not observe any hallucinations at ten-shots.

We report consistently low BLEU scores and high Levenshtein distances across zero- and few-shot learning for Gemma-7b-IT. The output was grossly hallucinatory, including hallucinated medical history (“historical factors include prior trauma-related injury sustained one month back”), translations into other languages, and treatment recommendations (“she’ll have an appointment to see her Dr tomorrow so we can discuss it then”). We therefore did not include a further evaluation of recall for individual PII categories for Gemma-7b-IT.

#### PII redaction per category

Names and dates were redacted consistently by all models (Table 3). However, medical record numbers, phone numbers, and the other unique identifiers had variable redaction across models. The Microsoft Azure de-identification service, OBI RoBERTa and BERT models, GPT-4-0125, Llama-3-8B-Instruct, and Phi-3-mini-128k-instruct had consistently high recall across PII categories.

**Table 3:** Recall per PII category

Model type	Model name	Names	Dates	Medical record numbers	Phone numbers
Proprietary de-identification software	Microsoft Azure de-identification service	0.976 (0.972–0.981)	0.981 (0.975–0.986)	0.913 (0.896–0.929)	0.972 (0.954–0.988)
	AnonCAT- base model	0.981 (0.977–0.985)	0.824 (0.804–0.844)	0.528 (0.498–0.560)	0.236 (0.177–0.295)
	AnonCAT- adapted & fine-tuned	0.785 (0.774–0.797)	0.954 (0.944–0.963)	0.911 (0.892–0.928)	0.987 (0.973–0.997)
	obi/deid_roberta_i2b2	0.979 (0.976–0.982)	0.972 (0.967–0.976)	0.924 (0.912–0.935)	0.994 (0.988–0.999)
	obi/deid_bert_i2b2	0.963 (0.958–0.967)	0.933 (0.925–0.941)	0.909 (0.896–0.921)	0.958 (0.942–0.973)
Large language models	Llama-3-8B-Instruct	1.00 (1.00–1.00)	0.980 (0.963–0.993)	0.989 (0.971–1.000)	1.000 (1.000–1.000)
	Phi-3-mini-128k-instruct	0.978 (0.969–0.985)	0.940 (0.926–0.953)	0.973 (0.961–0.983)	0.983 (0.963–0.997)
	GPT-3.5-turbo-0125	0.911 (0.893–0.928)	0.882 (0.860–0.902)	0.866 (0.840–0.890)	0.935 (0.900–0.967)
	GPT4-0125	0.988 (0.983–0.992)	0.970 (0.960–0.979)	0.966 (0.953–0.977)	0.994 (0.986–1.000)

Results for AnonCAT are shown for fine-tuning with 365 examples; results for LLMs are shown using 10 shot learning.

The base AnonCAT model performed poorly on redacting phone numbers (recall 0.236; 0.177–0.295), but following fine-tuning improved to a near-perfect sensitivity (0.987; 0.973–0.997). Qualitative analysis revealed that this was due to differences in the format of internal hospital phone extensions and bleep numbers, which was learned during the tuning process. We observed an unexpected decrease in recall for names after introducing new concepts and performing fine-tuning, possibly indicating some loss of earlier learning (catastrophic forgetting).

#### PII redaction per dataset

**Figure 2:**
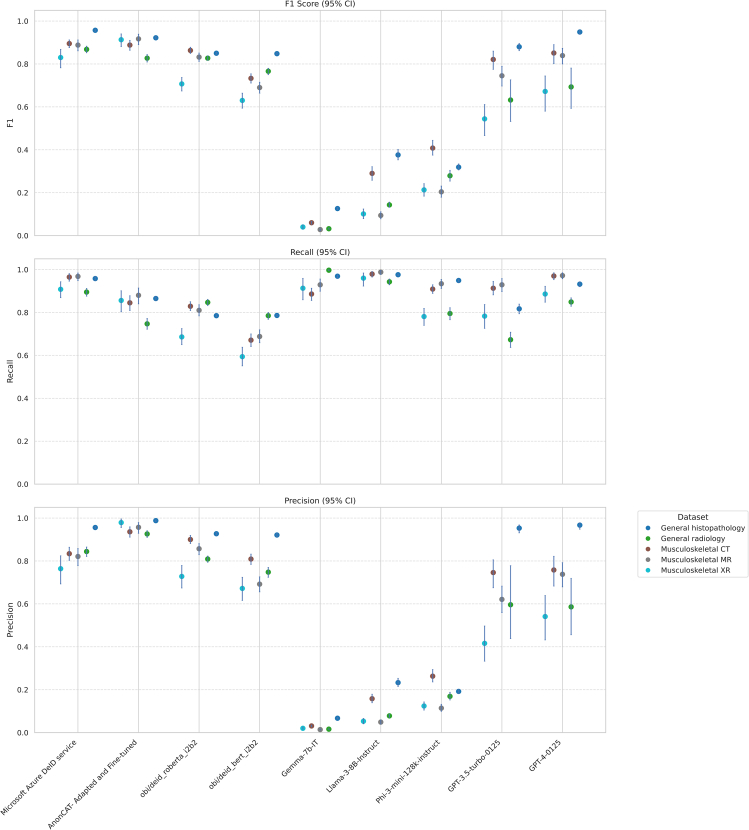
Model performance per dataset, with 95% confidence intervals All LLM results are plotted using performance with ten shots. Panel A shows F1 score, panel B recall, and panel C precision. Data are represented as mean ± 95% confidence intervals.

Of the best performing models, the adapted AnonCAT model showed the least performance shift between dataset, followed by the Microsoft Azure de-identification service (Figure 2; Table S4). GPT-4-0125, the best performing LLM, had a wide range of performance across datasets, with highest performance in the general histopathology dataset, with an F1 0.949 (0.938–0.958), and lowest in the musculoskeletal radiograph dataset, with an F1 0.672 (0.580–0.744). Likewise, GPT-3.5-turbo-0125, Llama-3-8B-Instruct, and Phi-3-mini-128k-Instruct had more performance shift across datasets than both task-specific models.

## Discussion

In this study, we evaluated the performance of four task-specific de-identification models and five “general-purpose” LLMs in de-identification of unstructured and semi-structured clinical text, using a dataset of 3,650 individual records. Our results suggest that automated de-identification, using either purpose-built software or foundational LLMs, have performance approaching that of human clinicians.

Recent studies have explored the capabilities of general-purpose LLMs for clinical text de-identification. Altalla et al. (2025), evaluates the capabilities of zero-shot GPT-3.5 and GPT-4 models in de-identifying 100 discharge summaries from a single center in Jordan, finding high F1, precision, and recall (0.897, 0.993, and 0.832, respectively) in GPT-4.^22^ Meaney et al., (2022) evaluate the performance of fine-tuned transformer models, including BERT-large and ROBERTA-large, on the i2b2 2014 corpus, finding differential performance across PII categories.^19^^,^^23^ Gunay et al. (2024), introduced an “LLM-in-the-loop” methodology, using LLMs to generate synthetic data, perform evaluation and agent orchestration in service of smaller, domain specific, Named Entity Recognition models, achieving high micro-F1 scores (>0.953) using the i2b2 2014 benchmark dataset.^19^^,^^24^ Our study complements and extends previous work by comparing task-specific models, with zero-shot, few-shot, and fine-tuned LLMs using a UK dataset. We perform a granular assessment of domain shift and model generalizability in a distinct healthcare setting and with different document types.

Our findings illustrate that purpose-built redaction models, and LLMs with larger (>8bn) parameter spaces supported by a few in-context examples, provided effective strategies for clinical redaction approaching near-human performance. Where supported, adaptation of models, including fine-tuning of task-specific models and in-context learning of LLMs, commonly improved redaction performance. The Microsoft De-identification service achieved the highest performance in our study, despite not presently supporting site-specific adaptation. The performance of AnonCAT was significantly improved by fine-tuning and addition of concepts to align our study ontology with the model’s default redaction behavior. This customizable architecture offers significant strengths where a hospital’s redaction needs differ, similar to promptable, generalist LLMs.

The best-performing LLMs in this study de-identified clinical notes with high performance using zero- or few-shot learning, suggesting that it is feasible to avoid or reduce the burden of curating large, manually labeled datasets for model finetuning. However, in all models, we report performance shift across domains. In general, domain shift was most pronounced between general and specialized, and semi-structured and unstructured reports. For example, GPT-4-0125 achieved an F1 score of 0.949 on general histopathology reports but only 0.672 on musculoskeletal radiograph reports. In our dataset, histopathology reports are templated, and consistently formatted identifiers, such as “specimen identifier” (see Supplement). In contrast, musculoskeletal imaging reports are unstructured, and include domain-specific terminology such as eponymous fracture patterns. Our findings underscore the importance of evaluating de-identification models across diverse clinical domains, to ensure robustness in real-world deployment.

There was variable performance across LLMs. Both Phi-3-mini-128k-instruct and Llama-3-8B-IT over-redacted records, reflected by consistently high recall in all domains and across all datasets but low precision. Qualitative examination revealed that clinically relevant information was redacted incorrectly; for example, diagnoses or symptoms, reducing the downstream utility of the redacted records. Here, Gemma-7b-IT was particularly prone to hallucination. Qualitative examination showed a mixture of hallucinations, ranging from explaining the task to offering clinical recommendations. We also used BLEU scores and Levenshtein distance as proxy indicators of hallucination, observing that low BLEU or high Levenshtein values often coincided with fabricated content. While these outputs may not pose a direct re-identification risk, they may compromise the interpretability and integrity of clinical documents when used for downstream research. Importantly, hallucinations were not observed in larger models, such as GPT-4-0125, particularly when provided with structured prompts and in-context examples. Additionally, increasing LLM parameter size correlated with improved performance, as demonstrated through the superior zero- and few-shot performance characteristics of the GPT-series models.

However, both human and model-based redaction, while good, is not perfect. A single clinician identified 98.6% of all PII, with 96.7% of words redacted representing true PII. The best performing tool overall, Microsoft’s de-identification service identified 95% of PII, including 97.6% of all names, and 92.8% of words redacted represented true PII.

A fundamental issue is what constitutes acceptable performance. Although attractive to strive for perfect redaction, the human performance and inter-annotator variability we describe shows this is unlikely to be possible without significant extra resource. One approach is to examine existing precedent. The MIMIC datasets are among the largest publicly available datasets to date. These are redacted by a combination of removing all entries from a database of identifiers, e.g., names, as well as by other string-pattern matching. The recall of this approach on a test corpus was 0.943.^10^ Similar performance was achieved by the Microsoft Azure de-identification service, adapted AnonCAT, and GPT-4-0125 in our analysis, strengthening confidence in the cross-national generalizability of included models. Future work could include hybrid approaches based on databases of identifiers, on a population, institution or personal level, to enhance redaction of these alongside generic model-based approaches. Additionally, to better understand the practical implications of imperfect redaction, future research should consider evaluating de-identification performance in the context of re-identification risk, for example, simulating realistic attack scenarios, such as linking redacted records with publicly available data, or exploring adversarial approaches to test model robustness. Our evaluation focuses on text de-identification, but this must be considered within a broader privacy strategy. Privacy-preserving strategies also include policy and governance controls, use of trusted research environments, access controls and audit, and privacy preserving computation such as secure enclaves.

### Limitations of the study

Our study has several limitations. While our study assessed token-level redaction performance, we did not explicitly evaluate the residual risk of re-identification, which depends not only on model performance but also on the nature of the retained text and the contextual uniqueness of PII. The data from this study derived from a single hospital group in the United Kingdom, and model performance may vary across sites. Our dataset comprises only English-language clinical notes, and we cannot comment on the performance of automated de-identification in other languages. All LLMs in this study were pretrained on an English-dominant corpus, and may transfer differentially to other languages, particularly languages from low-resource settings.^25^^,^^26^^,^^27^ More extensive adaptation of LLMs such as fine-tuning, as shown for the task-specific AnonCAT, may improve redaction performance particularly where data formats differ significantly between sites. However, this would entail considerable computational expense and technical burden and presently is not amenable to being readily deployable. Fine-tuning a general purpose LLM for de-identification, on a few-hundred to a few-thousand labeled notes requires access to a modern GPU and modest engineering support. Once trained, inference is fast and can be hosted on-premise. Zero- and few-shot LLM use removes the need for training and can work well from the first day, at the cost of sending prompts and notes to an external endpoint unless an on-premise model is available.

Operational latency and deployment costs, through critical practical considerations, were not explicitly measured in this evaluation. Latency varies substantially based on hardware and software optimization for on-premise models, while cloud-hosted solutions typically offer low-latency inference. Cost structures also differ significantly: cloud-based solutions involve usage-based pricing, which changes with time and volume of data, whereas on-premise deployment includes upfront hardware investment, ongoing maintenance costs, and cost of fine-tuning. Task-specific models are commonly packaged for hospital servers and air-gapped environments, and some vendors support concept set alignment and full fine-tuning at cost. Cloud-hosted LLMs offer immediate availability but require information governance and engineering expertise to prevent data leaks and may be restricted depending on locality. For clinical users and researchers, the most convenient option is a single drop-in service that can take heterogenous documents and return the redacted versions. When local formats differ, light adaptation also works well: for LLMs, a small set of site-specific examples improves performance. Such practical factors should be evaluated alongside accuracy when considering modes for clinical implementation.

We were unable to evaluate redaction of uncommon, but critical, categories of PII; e.g., email addresses. Furthermore, we were unable to comment on sub-categories of PII, including specific performance for patient names, due to the low prevalence in our dataset. We qualitatively examined 50 examples of output for each model, per-shot, for hallucination, but cannot conclusively report on the prevalence of hallucination for the entire dataset. Finally, we report on seven different comparators, but since conducting the analyses, other software or LLMs may have been developed with superior performance.

If redaction is imperfect, other protections are required to protect privacy and confidentiality. These include requiring researchers to not attempt re-identification of individuals, to only access sufficient data to answer research questions, and retaining data within secure data environments, limiting access to approved researchers. Acceptability of research continues to require multi-stakeholder representation, and is preconditioned on ethical conduct beyond simply de-identification of patient records.

Within the scope of these protections, automating de-identification of clinical notes could enable large-scale sharing of clinical data and its use in training large-scale models, with less time and expense than manual de-identification. Quality assurance through careful examination of model output, across clinical and PII domains, with attention to hallucinatory output, and loss of critical information through over-redaction is crucial in real-world practice. Hallucinations, particularly those that fabricate clinical information, pose a non-trivial risk to the integrity of downstream research. We suggest future research focusing on systematic, scalable techniques to detect and suppress hallucinations, especially in zero- and few-shot scenarios. Agentic approaches may offer promising avenues for future research, for example, by introducing an ensemble or error-checking mechanisms, with the caveats of increased computational expense.

In summary, our results support the use of automated de-identification systems using no- or low-adaptation strategies. We recommend care and quality assurance in deployment, particularly in implementing systems based on general-purpose LLMs.

## Resource availability

### Lead contact

Requests for further information and resources should be directed to and will be fulfilled by the lead contact, Rachel Kuo (rachel.kuo@ndorms.ox.ac.uk).

### Materials availability

The datasets analyzed during the current study are not publicly available as they contain personal data. The histology and general radiology data are available from the Infections in Oxfordshire Research Database (https://oxfordbrc.nihr.ac.uk/research-themes/modernising-medical-microbiology-and-big-infection-diagnostics/iord-about/), subject to an application and research proposal meeting the ethical and governance requirements of the Database. For further details on how to apply for access to the data and a research proposal template please email iord@ndm.ox.ac.uk. Example, synthetic data are provided in the Supplement, for illustration.

### Data and code availability

• The data analyzed in this study are not publicly available as they contain personal data.
• All code used in this work, and a small synthetic dataset generator, are open-source at https://github.com/andrewsoltan/ehr-deidentification-for-health/.

## Acknowledgments

This work uses data provided by patients and collected by the UK’s National Health Service as part of their care and support. We thank all the people of Oxfordshire who contribute to the Infections in Oxfordshire Research Database. Research Database Team: L. Butcher, H. Boseley, C. Crichton, D.W. Crook, D.W.E., O. Freeman, J. Gearing (community), R. Harrington, K. Jeffery, M. Landray, A. Pal, T.E.A. Peto, T.P. Quan, J. Robinson (community), J. Sellors, B. Shine, A.S. Walker, and D. Waller. Patient and Public Panel: M. Ahmed, G. Blower, J. Hopkins, V. Lekkos, R. Mandunya, S. Markham, and B. Nichols.

Funding: R.K. is supported by a 10.13039/501100000272National Institute for Health Research (NIHR) Doctoral Research Fellowship (NIHR302562). A.S. is supported by the Microsoft Accelerating Foundation Models (10.13039/100003578AFMR) grant; compute costs for this study, of $1000, were funded by the 10.13039/100003578AFMR award. D.A.C. is supported by the Pandemic Sciences Institute at the 10.13039/501100000769University of Oxford; the 10.13039/501100000272National Institute for Health Research (NIHR), Oxford Biomedical Research Centre (BRC); an NIHR Research Professorship; a Royal Academy of Engineering Research Chair; the 10.13039/100010269Wellcome Trust funded VITAL project (grant 204904/Z/16/Z); the 10.13039/501100000266EPSRC (grant EP/W031744/1); and the InnoHK Hong Kong Centre for Cerebro-cardiovascular Engineering (COCHE). D.W.E. is a Robertson Foundation Fellow. This study was also supported by the NIHR Health Protection Research unit in Healthcare Associated Infections and Antimicrobial Resistance at Oxford University in partnership with the UK Health Security Agency (UKHSA) and the 10.13039/501100013373NIHR Biomedical Research Centre, Oxford. The views expressed in this publication are those of the authors and not necessarily those of the NHS, the 10.13039/501100000272National Institute for Health Research, the 10.13039/501100000276Department of Health and Social Care or the UKHSA. We would like to thank Kimia Mavon (Microsoft Research UK) for provision of the Microsoft Azure de-identification service, and Richard Dobson and Xi Bai (CogStack, and Kings College London) for provision of AnonCAT free-of-charge for the purposes of this research. Neither party and none of the funders had input into study design, data collection, data annotation, data analysis, interpretation of results, or writing of the manuscript.

## Author contributions

R.K. and A.A.S.S. were equally responsible for conceptualization, methodology, software, validation, formal analysis, investigation, resources, writing, project administration, funding acquisition, and visualization. R.K., C.O.’H., and A.H. performed data curation. A.A.S.S. hosts the code base on GitHub. D.A.C. and G.C. provided input into methodology, validation, and supervision. D.F. and D.W.E. equally contributed to the conceptualization, methodology, resources, writing, project administration, funding acquisition and supervision of this study.

## Declaration of interests

D.A.C. reports personal fees from Oxford University Innovation, outside the submitted work. No other author has a conflict of interest to declare.

## STAR★Methods

### Key resources table

**Table:** 

REAGENT or RESOURCE	SOURCE	IDENTIFIER
**Software and algorithms**		

Microsoft Azure de-identification (DeID) service	https://techcommunity.microsoft.com/t5/healthcare-and-life-sciences/announcing-a-de-identification-service-for-health-and-life/ba-p/3949712	N/A
AnonCAT	Kraljevic Z et al.^28^	N/A
OBI RoBERTa & BERT i2b2 DeID Models	Kailas et al.^29^	N/A
Gemma-7b-IT	Team G et al.^30^	N/A
Llama-3-8B-Instruct	Zhao J et al.^25^	N/A
Phi-3-mini-128k-instruct	Abdin M et al.^31^	N/A
GPT-3.5-turbo-0125 and GPT4-turbo-0125	Achiam J et al.^26^	N/A

**Code**		

Code	https://github.com/andrewsoltan/ehr-deidentification-for-health/	

### Experimental model and study participant details

#### Human participants and data sources

We used routinely collected data from Oxford University Hospitals NHS Foundation Trust (OUHNFT), a large teaching trust comprised of four hospitals providing care to around 1% of the UK population as well as specialist referral services.^32^ Data were obtained from two sources between January-2020-January-2022 inclusive. We randomly sampled 1000 general radiology and 1000 histopathology reports from the Infections in Oxfordshire Research Database (IORD). These records include all inpatient and outpatient medical specialties, and comprise a general dataset. IORD has approvals from the National Research Ethics Service South Central – Oxford C Research Ethics Committee (19/SC/0403), the Health Research Authority and the Confidentiality Advisory Group (19/CAG/0144). Structured identifiers were removed from records prior to analysis, but free-text was provided without further redaction to researchers with NHS contracts for analysis within NHS infrastructure. We also randomly sampled 550 radiograph, 550 computed tomography (CT), and 550 magnetic resonance (MR) request and report entries from a specialised musculoskeletal database, with Camden and Kings Cross Research Ethics Committee approval (22/LO/0049). The specialised musculoskeletal dataset focusses on the upper limb. This subspecialty is a minority representation in existing benchmarks, which commonly centre on chest or neuroimaging, and provides a distinct domain with different structural and lexical characteristics, such as eponymous conditions. Together, the general and specialised datasets enable evaluation of model performance across clinical domains, with varying degrees of structure, PII prevalence, and vocabulary.

### Method details

#### PII definition and annotation rules

We developed our labelling ontology based on HIPAA PII categories,^21^ including three further categories we considered additional potential identifiers. These were: hospital/unit names, private healthcare organisation names, and individual professional details where included as part of a healthcare professionals’ name or where related to patients (Table 1). We excluded two inapplicable HIPAA categories (biometric identifiers and full-face photographic images).

**Table 1:** Categories of PII

	PII category	PII subcategory
1	Names	Patient or relative
		Healthcare worker
2	Address	House or street number
		Street name
		City
		County
		Country
		Postcode
3	Dates	Dates
		Age over 89 years
4	Phone numbers	–
5	Fax numbers	–
6	Electronic email addresses	–
7	Social security numbers	–
8	Medical record numbers	Medical record number
		NHS number
		Specimen identifier
9	Health plan beneficiary numbers	–
10	Account numbers	–
11	Certificate/license numbers	GMC number
		NMC number
		Any other license
12	Vehicle identifiers and serial numbers	–
13	Device identifiers and serial numbers	–
14	Web Universal Resource Locators (URLs)	–
15	Internet Protocol (IP) address numbers	–
16	Any other unique identifying number, characteristic, or code	Hospital/unit
		External healthcare organization
		Professional details

All examples were annotated by two clinically qualified labellers (two of RK, CO, and AH, details in Supplement), using the open-source software *doccano*, hosted on an OUHNFT server.^33^ Annotations were applied on a word-level and included PII category, and the start and end position of each annotation. After completion of labelling, any disagreements were recorded, and then resolved with discussion. This consensus annotation by clinicians was considered the ground truth for subsequent model evaluation.

#### Model selection and setup

We evaluated models using no, or low-adaptation approaches for de-identification. By no-adaptation, we refer to models used exactly as provided by their developers, without modification. Low-adaptation includes lightweight modifications such as prompt-based few-shot learning, or concept list adaptation for task-specific models.

Our evaluation included four purpose-built de-identification software based upon the transformer architecture (Microsoft Azure de-identification service, AnonCAT, One Brave Idea [OBI] RoBERTa & BERT i2b2 Models^28^^,^^34^) and five LLMs (Gemma-7b-IT, Llama-3-8B-Instruct, Phi-3-mini-128k-instruct, GPT-3.5-turbo-0125 and GPT4-0125^28^^,^^30^^,^^31^^,^^34^^,^^35^; all details in Supplement).

For each LLM, we compared performance with zero, one-, five- and ten-shot learning, primed using examples sourced from outside the dataset (Supplement). Task-specific models were evaluated as provided by the manufacturers (Supplement). All 3650 examples were used for evaluating performance.

To assess the change in performance brought about by adaptation of one tuneable task-specific model, we evaluated AnonCAT both using its default weights and updated weights following concept-expansion and fine-tuning. A randomly selected sample of 365 (10%) clinical notes stratified by dataset was used for tuning with a learning rate of 0.00002 over 10 epochs (Supplement), and the tuned-model was evaluated used the remaining 3285 documents.

We considered that some, but not all, uses of secondary data would necessitate the redaction of professional titles where this was related to healthcare professionals. The default behaviours of the Microsoft Azure de-identification service and OBI models was to redact professional titles. However, by default, the AnonCAT model does not redact professional titles and we therefore performed additional analyses of the untuned model to report performance without the requirement to redact professional titles. During adaptation, we expanded the concept set of the model to align AnonCAT’s base ontology with our own (adding concepts for professional titles, external healthcare organisation, hospital/unit, age over 89, account, URL, GMC number, and social security number). We therefore applied the requirement to redact professional titles only to the tuned model, i.e., similar to all other models in this study.

All inference, training, and analyses were performed on a virtual machine hosted on OUHNFT’s Azure tenancy between April and June 2024.

#### Prompt structure

Optimising LLM prompts improves downstream task performance.^36^ We provided each LLM with a structured prompt. First, we specify the task (‘Anonymise the following text’). Second, we specify rules for task completion (’Replace all identifiers with their classification’). Third, we provide the labelling ontology, and a brief, synthetic example of desired output (‘If you find a doctor’s name, such as ‘Dr John Doe’, replace this with [profession] [doctor] [doctor]’). Finally, we describe undesirable output (‘Do not output any additional text’).

#### Evaluation metrics, quantification and statistical analysis

We estimate word-level inter-annotator reliability of clinicians using precision (positive predictive value of a PII label), recall (sensitivity, the proportion of all PII detected) and pairwise F1 scores (harmonic mean of precision and recall), using two prediction classes (PII/non-PII).^37^ To assess variation between clinician-performed de-identification, the most senior clinician (RK) was chosen as the gold standard, and precision/recall were calculated using this assumption. The calculation of pairwise F1 score is independent of the designation of annotators as the gold standard or experimental source.^37^

Model performances were compared to a composite reference standard combining two clinician annotations; reporting precision, recall and F1 score for two prediction classes (PII/non-PII).

To describe per-category recall, we used the clinician-assigned PII categories as a reference and considered the PII to be correctly identified if it was redacted under any model-generated label. This reflects that some PII may be correctly classified in two categories, that proprietary models had different labelling ontologies, and redaction rather than PII classification is most important in real-world settings. We calculate recall for all categories comprising >1% of all PII in the dataset.

We defined LLM hallucinations as additional text that was not present in the reference text. As a proxy, we calculated BLEU scores and Levenshtein distance, commonly used metrics for measuring string similarity.^38^^,^^39^ The Levenshtein distance is defined as the minimum number of character-edits required to transform one string to another; here, from redacted to reference records.^38^ BLEU scores are defined by *n*-gram overlap between two strings; here, we calculated the cumulative 4-gram BLEU score.^39^ Low BLEU scores and high Levenshtein distances are potentially indicative of LLM hallucination. We qualitatively examined 50 randomly selected samples of each model output. We calculated 95% confidence intervals (CI) using bootstrapping with 10,000 samples. We coded all analyses using Python (v3.8.10).
