# Supplementary material for: Benchmarking transformer-based models for medical record de-identification in a single center multi-specialty evaluation
Source: iScience. 2025 Oct 8;28(12):113732. doi: 10.1016/j.isci.2025.113732 (PMC12719064; doi:10.1016/j.isci.2025.113732)
Supplement: Document S1. Figure S1, Tables S1–S4 and Methods S1 [file mmc1.pdf]

iScience, Volume *28*

Supplemental information

Benchmarking transformer-based models for medical record de-identification in a single center multi-specialty evaluation

Rachel Kuo, Andrew A.S. Soltan, Ciaran O'Hanlon, Alan Hasanic, David A. Clifton, Gary Collins, Dominic Furniss, and David W. Eyre

Comparative evaluation of large-language models and purpose-built software for medical record de-identification: Supplementary Material

Comparative evaluation of large-language models and purpose-built software for medical record de-identification: Supplementary Material 1

Supplementary methods 2

Software and models tested 2

AnonCAT fine-tuning 3

Supplementary tables 4

Table S1. Description per dataset 4

Table S2. Frequency of PHI categories within the dataset 6

Table S3. Per model results for classification of PII vs. non-PII. 8

Table S4. Model precision, recall and F1 score per dataset 9

Supplementary figures 11

Figure S1. LLM performance by shot 11

Methods S1. Supplementary methods

Software and models tested

De-identification software

Microsoft Azure de-identification service

The Microsoft Azure de-identification service is a paid-for, purpose-built clinical data de-identification pipeline, based on redacting PII using HIPAA categories[1]. The service is accessed within Azure Health Data Services and Microsoft Fabric and can be applied to unstructured or semi-structured text.

AnonCAT

AnonCAT is a transformer-based (RoBERTa-large) model, purpose-built for de-identification of unstructured clinical data[2]. It was trained using 2648 manually annotated clinical documents from King's College Hospital NHS Foundation Trust in the UK. Two smaller sets from Guy's and St Thomas' NHS Foundation Trust (328 documents) and University College London Hospitals NHS Foundation Trust (140 documents) were used for fine-tuning and testing the model. AnonCAT is offered as a paid-for service; the core library and annotation tools are open source.

Large language models

Gemma-7b-IT

The Gemma models are a family of open-source LLMs, trained on 6T tokens of text, based on Google's *Gemini* models[3]. For this study, we evaluated Gemma-7b-IT, which is a 7 billion parameter model.

Llama-3-8B-Instruct

The Meta Llama 3 family of LLMs are open-source, auto-regressive language models that use transformer architecture. The model evaluated in this study has 8 billion parameters and is instruction tuned. Information regarding model training and testing are not available via publication.

Phi-3-mini-128k-instruct

Phi-3-mini is an open-source, 3.8 billion parameter transformer-decoder model, trained on 3.3 trillion tokens, provided by Microsoft[4]. In this study, we evaluated Phi-3-mini-128k-instruct.

GPT3.5-turbo-base and GPT4 turbo

GPT3.5 and GPT4 are paid-for, transformer based LLMs developed by OpenAI[5]. Information regarding model architecture, training and testing are not available via publication.

LLM hyperparameters

To assess LLM output for zero- and few-shot learning, we used the following hyperparameters: 0, one, five and ten-shots; 3000 was set as the maximum number of output tokens, temperature 0.1, top-k 50 and top-p 0.95. We selected these hyperparameters to increase the predictability and conservativeness of LLM output, given the nature of the task.

OpenAI models (GPT-3.5-turbo-base and GPT-4-0125) were used via the Azure OpenAI service, which is an enterprise grade service that does not retain prompt data for training or service improvement.

AnonCAT fine-tuning

We fine-tuned AnonCAT using 365 (10%) randomly selected documents from our annotated data, stratified by dataset. We split the fine-tuning dataset into training (292, 80%) and evaluation (73, 20%) sets. As the ontology of AnonCAT de-identification was not based on HIPAA, we manually coded our labelling ontology to the equivalent categories. For example, ‘GMC’ was coded to ‘H3500’ or ‘Healthcare professional’.

We set a learning rate of 0.000002 over 6 epochs, reporting a final validation loss of 0.06166.

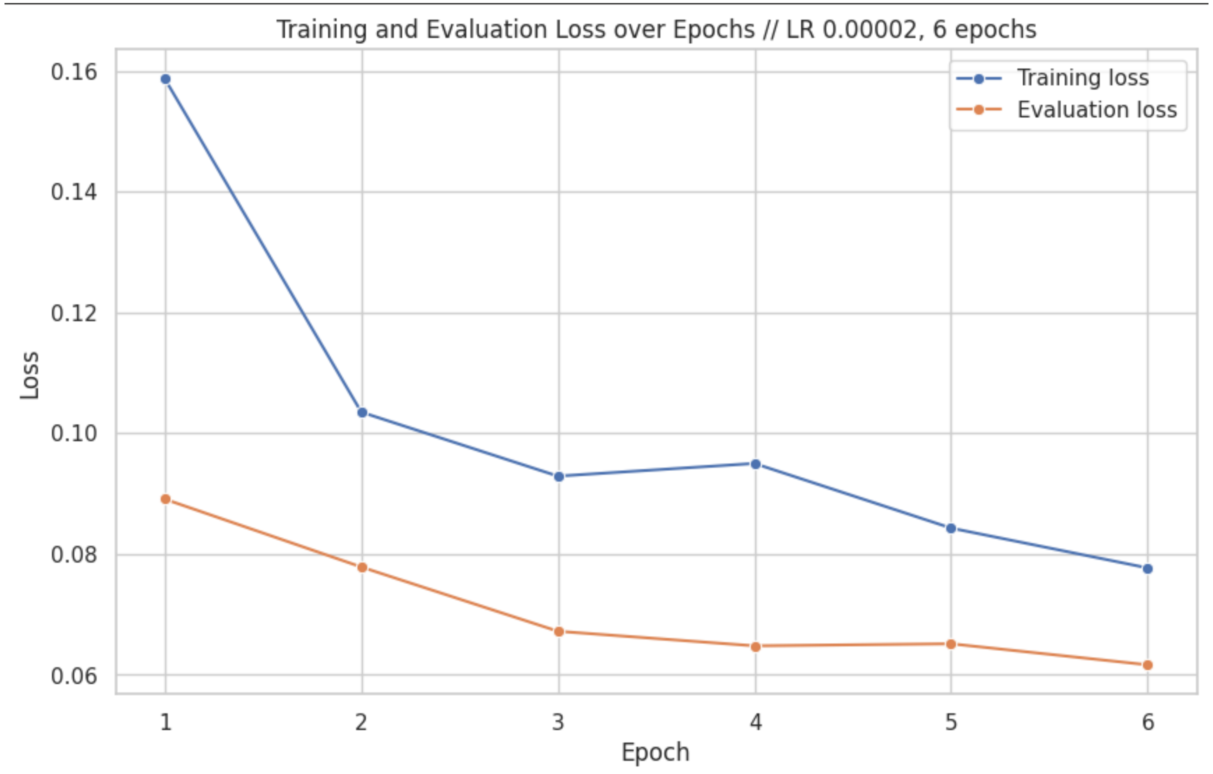

Figure S1. AnonCAT fine-tuning: training and evaluation set loss, over epochs

Supplementary tables

Table S1. Description per dataset

Dataset	Records	Total words	Mean words (IQR)	Prevalence of PHI (%)
Musculoskeletal XR	550	25549	46.45 (30-57)	272 (1.06%)

Musculoskeletal CT	550	40920	74.40 (44-94)	688 (1.68%)
Musculoskeletal MR	550	55513	100.93 (58-132)	452 (0.81%)
General radiology	1000	134385	134.49 (61-183)	1803 (1.34%)
General histopathology	1000	223393	223.39 (123-262)	14281 (6.39%)

Table S2. Frequency of PHI categories within the dataset

PHI category by frequency	PHI subcategory	Count (% of PHI)
Names	**Total**	**6901 (39.44%)**
	Patient or relative	31
	Healthcare professional name	6870
Any other unique identifying number, characteristic, or code	**Total**	**4758 (21.19%)**
	Hospital/unit	570
	External healthcare organisation	1037
	Professional details	3151
Dates	**Total**	**3641 (20.81%)**
	Date	3628
	Age over 89 years old	13
Medical record numbers	**Total**	**1408 (8.05%)**
	Medical record number	22
	NHS number	4
	Specimen identifier	1382
Phone numbers	**Total**	**334 (1.91%)**
Address	**Total**	**133 (0.76%)**
	House or street number	35
	Street name	32
	City	27
	County	5
	Country	18
	Postcode	16
Web Universal Resource Locators (URLs)	**Total**	**144 (0.82%)**
Certificate/license numbers	**Total**	**107 (0.61%)**
	GMC number	103
	NMC number	0

	Any other license	4
Electronic email addresses	**Total**	**59 (0.34%)**
Account numbers	**Total**	**10 (0.06%)**
Social security numbers	**Total**	**1 (0.01%)**

Table S3. Per model results for classification of PII vs. non-PII.

Model type	Model name	Number of shots	Precision (95% CI)	Recall (95% CI)	F1 (95% CI)
**Inter-annotator**		**N/A**	**0.967 (0.932-0.993)**	**0.986 (0.971-0.997)**	**0.977 (0.957-0.991)**
Proprietary de-identification software	Microsoft Azure de-identification service	N/A	0.916 (0.910-0.922)	0.950 (0.942-0.957)	0.933 (0.928-0.938)
	AnonCAT	No fine-tuning, without requirement to redact professional details	0.937 (0.930-0.943)	0.778 (0.767-0.790)	0.850 (0.843-0.858)
		No fine-tuning, with requirement to redact professional details	0.937 (0.930-0.943)	0.668 (0.659-0.676)	0.780 (0.773-0.786)
		Fine-tuned, without requirement to redact professional details	0.981 (0.977-0.985)	0.787 (0.773-0.800)	0.873 (0.864-0.882)
		Fine-tuned, with requirement to redact professional details	0.981 (0.977-0.985)	0.676 (0.665-0.686)	0.800 (0.843-0.858)
Large language models	Gemma-7b-IT	0	0.047 (0.045-0.048)	0.870 (0.859-0.880)	0.089 (0.086-0.092)
		1	0.042 (0.040-0.043)	0.941 (0.933-0.948)	0.080 (0.077-0.083)
		5	0.043 (0.042-0.045)	0.974 (0.969-0.978)	0.083 (0.080-0.086)
		10	0.021 (0.019-0.023)	0.905 (0.885-0.923)	0.041 (0.037-0.044)
	Llama-3-8B-Instruct	0	0.041 (0.037- 0.045)	0.943 (0.928- 0.956)	0.079 (0.072- 0.087)
		1	0.048 (0.043-0.052)	0.984 (0.976-0.991)	0.091 (0.083-0.010)
		5	0.110 (0.099-0.121)	0.990 (0.983-0.995)	0.198 (0.181-0.216)
		10	0.077 (0.069-0.085)	0.978 (0.969-0.987)	0.143 (0.130-0.157)
	Phi-3-mini-128k-instruct	0	0.080 (0.076-0.084)	0.829 (0.814-0.845)	0.146 (0.140-0.153)
		1	0.155 (0.145-0.166)	0.820 (0.804-0.835)	0.261 (0.246-0.276)
		5	0.121 (0.115-0.127)	0.904 (0.892-0.915)	0.213 (0.204-0.222)

		10	0.297 (0.282-0.314)	0.904 (0.892-0.915)	0.448 (0.430-0.467)
	GPT3.5-turbo-base	0	0.388 (0.371-0.405)	0.838 (0.822-0.854)	0.530 (0.514-0.547)
		1	0.476 (0.455-0.496)	0.767 (0.749-0.784)	0.588 (0.570-0.604)
		5	0.764 (0.725-0.796)	0.761 (0.745-0.777)	0.763 (0.741-0.781)
		10	0.856 (0.812-0.892)	0.807 (0.788-0.825)	0.831 (0.807-0.851)
	GPT-4-0125	0	0.825 (0.789-0.853)	0.930 (0.920-0.940)	0.874 (0.853-0.891)
		1	0.814 (0.774-0.848)	0.931 (0.920-0.941)	0.868 (0.845-0.888)
		5	0.839 (0.797-0.875)	0.931 (0.921-0.940)	0.883 (0.858-0.903)
		10	0.874 (0.834-0.906)	0.924 (0.914-0.933)	0.898 (0.876-0.916)

Table S4. Model precision, recall and F1 score per dataset

Model type	Model name	Number of shots	Dataset	Precision (95% CI)	Recall (95% CI)	F1 (95% CI)
Proprietary de-identification software	Microsoft Azure de-identification service	N/A	Musculoskeletal XR	0.710 (0.651-0.761)	0.893 (0.854-0.930)	0.791 (0.749-0.825)
			Musculoskeletal CT	0.818 (0.787-0.847)	0.965 (0.946-0.981)	0.886 (0.866-0.903)
			Musculoskeletal MR	0.787 (0.747-0.824)	0.968 (0.949-0.983)	0.868 (0.841-0.892)
			General radiology	0.825 (0.802-0.845)	0.894 (0.875-0.911)	0.858 (0.842-0.873)
			General histopathology	0.948 (0.942-0.954)	0.958 (0.949-0.967)	0.953 (0.947-0.959)
	AnonCAT	No fine-tuning, without requirement to redact professional details	Musculoskeletal XR	0.955 (0.920-0.985)	0.627 (0.565-0.685)	0.757 (0.709-0.798)
			Musculoskeletal CT	0.971 (0.950-0.989)	0.615 (0.559-0.669)	0.753 (0.708-0.793)
			Musculoskeletal MR	0.955 (0.920-0.985)	0.627 (0.565-0.685)	0.757 (0.709-0.798)
			General radiology	0.892 (0.865-0.916)	0.590 (0.544-0.625)	0.710 (0.681-0.737)
			General histopathology	0.939 (0.932-0.947)	0.825 (0.812-0.838)	0.879 (0.870-0.887)
			Musculoskeletal XR	0.955 (0.920-0.984)	0.562 (0.508-0.616)	0.707 (0.663-0.749)

		No fine-tuning, with requirement to redact professional details	Musculoskeletal CT	0.971 (0.950-0.989)	0.573 (0.526-0.622)	0.796 (0.790-0.803)
			Musculoskeletal MR	0.973 (0.953-0.990)	0.668 (0.621-0.712)	0.792 (0.757-0.823)
			General radiology	0.892 (0.865-0.916)	0.565 (0.531-0.597)	0.691 (0.663-0.717)
			General histopathology	0.939 (0.932-0.947)	0.691 (0.682-0.699)	0.796 (0.790-0.803)
		Fine-tuned, without requirement to redact professional details	Musculoskeletal XR	0.958 (0.916-0.992)	0.640 (0.573-0.704)	0.767 (0.715-0.812)
			Musculoskeletal CT	0.955 (0.932-0.975)	0.735 (0.695-0.776)	0.830 (0.802-0.858)
			Musculoskeletal MR	0.957 (0.930-0.980)	0.756 (0.708-0.805)	0.845 (0.812-0.877)
			General radiology	0.932 (0.913-0.949)	0.654 (0.622-0.687)	0.769 (0.746-0.791)
			General histopathology	0.991 (0.987-0.994)	0.814 (0.798-0.831)	0.894 (0.883-0.904)
		Fine-tuned, with requirement to redact professional details	Musculoskeletal XR	0.958 (0.916-0.992)	0.572 (0.511-0.633)	0.717 (0.667-0.673)
			Musculoskeletal CT	0.955 (0.932-0.975)	0.735 (0.695-0.776)	0.830 (0.802-0.858)
			Musculoskeletal MR	0.957 (0.930-0.980)	0.674 (0.629-0.722)	0.791 (0.757-0.825)
			General radiology	0.932 (0.913-0.949)	0.626 (0.594-0.657)	0.749 (0.725-0.770)
			General histopathology	0.991 (0.987-0.994)	0.682 (0.670-0.694)	0.808 (0.799-0.816)
Large language models	Gemma-7b-IT	10	Musculoskeletal radiograph	0.020 (0.016-0.026)	0.913 (0.859-0.959)	0.040 (0.031-0.050)
			Musculoskeletal CT	0.031 (0.027-0.035)	0.886 (0.856-0.913)	0.060 (0.053-0.067)
			Musculoskeletal MR	0.014 (0.012-0.017)	0.929 (0.899-0.956)	0.028 (0.023-0.032)
			General radiology	0.016 (0.015-0.018)	0.997 (0.995-0.999)	0.032 (0.030-0.035)
			General histopathology	0.067 (0.065-0.070)	0.969 (0.962-0.975)	0.126 (0.121-0.131)
	Llama-3-8B-Instruct	10	Musculoskeletal radiograph	0.053 (0.041-0.066)	0.960 (0.923-0.984)	0.101 (0.079-0.124)
			Musculoskeletal CT	0.158 (0.139-0.179)	0.979 (0.965-0.991)	0.290 (0.258-0.321)
			Musculoskeletal MR	0.049 (0.040-0.059)	0.988 (0.977-0.996)	0.094 (0.078-0.111)
			General radiology	0.078 (0.071-0.085)	0.943 (0.929-0.956)	0.143 (0.132-0.156)

			General histopathology	0.233 (0.215-0.253)	0.976 (0.971-0.980)	0.376 (0.353-0.402)
	Phi-3-mini-128k-instruct	10	Musculoskeletal radiograph	0.124 (0.105-0.143)	0.781 (0.740-0.819)	0.213 (0.184-0.242)
			Musculoskeletal CT	0.263 (0.236-0.294)	0.909 (0.889-0.929)	0.408 (0.375-0.444)
			Musculoskeletal MR	0.114 (0.099-0.132)	0.934 (0.912-0.954)	0.204 (0.179-0.231)
			General radiology	0.169 (0.152-0.188)	0.795 (0.766-0.822)	0.279 (0.254-0.304)
			General histopathology	0.192 (0.182-0.202)	0.949 (0.941-0.956)	0.319 (0.306-0.334)
	GPT3.5-turbo-base	10	Musculoskeletal radiograph	0.416 (0.333-0.497)	0.783 (0.726-0.837)	0.544 (0.466-0.611)
			Musculoskeletal CT	0.746 (0.675-0.805)	0.913 (0.882-0.945)	0.821 (0.775-0.860)
			Musculoskeletal MR	0.621 (0.559-0.683)	0.929 (0.897-0.958)	0.745 (0.697-0.789)
			General radiology	0.596 (0.438-0.778)	0.673 (0.637-0.708)	0.632 (0.532-0.726)
			General histopathology	0.953 (0.931-0.968)	0.817 (0.794-0.839)	0.880 (0.862-0.896)
	GPT-4-0125	10	Musculoskeletal radiograph	0.541 (0.432-0.639)	0.886 (0.848-0.922)	0.672 (0.580-0.744)
			Musculoskeletal CT	0.758 (0.683-0.822)	0.970 (0.953-0.985)	0.851 (0.801-0.890)
			Musculoskeletal MR	0.738 (0.679-0.792)	0.972 (0.957-0.985)	0.839 (0.800-0.873)
			General radiology	0.586 (0.456-0.719)	0.849 (0.829-0.868)	0.693 (0.593-0.781)
			General histopathology	0.967 (0.948-0.978)	0.932 (0.920-0.943)	0.949 (0.938-0.958)

Supplementary figures

Figure S1. LLM performance by shot

Per LLM results for classification of PII vs. non-PII across few-shot learning

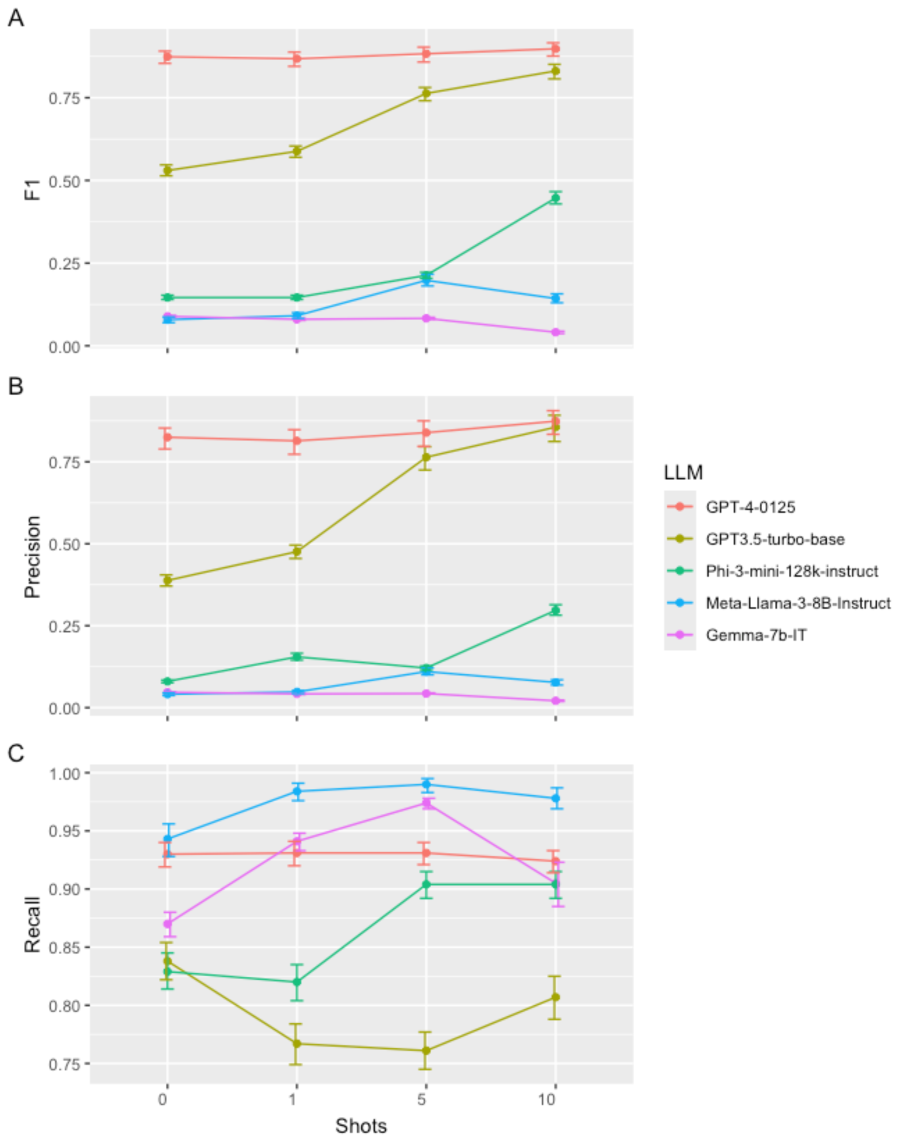

Supplementary references

1. Mavon K. Microsoft De-Identification Service. https://techcommunity.microsoft.com/t5/healthcare-and-life-sciences/announcing-a-de-identification-service-for-health-and-life/ba-p/3949712 Web site. . Accessed June, 2024

2. Kraljevic Z, Shek A, Yeung JA, et al. Validating transformers for redaction of text from electronic health records in real-world healthcare. 2023 IEEE 11th International Conference on Healthcare Informatics (ICHI)

3. Team G, Mesnard T, Hardin C, et al. Gemma: Open models based on gemini research and technology. *arXiv preprint arXiv:2403.08295.* 2024

4. Abdin M, Jacobs SA, Awan AA, et al. Phi-3 technical report: A highly capable language model locally on your phone. *arXiv preprint arXiv:2404.14219.* 2024

5. Achiam J, Adler S, Agarwal S, et al. Gpt-4 technical report. *arXiv preprint arXiv:2303.08774.* 2023
